# The GNAS Gene: Fibrous Dysplasia, McCune–Albright Syndrome, and Skeletal Structure and Function

**DOI:** 10.3390/genes16111360

**Published:** 2025-11-10

**Authors:** Jake Louis Littman, Wentian Yang, Noah Feder, Amr Kaadan, Ali Amin, Roy K. Aaron

**Affiliations:** 1 School of Medicine, University of Pittsburgh, Pittsburgh, PA 15213, USA; jal544@pitt.edu (J.L.L.); feder.noah@medstudent.pitt.edu (N.F.); 2 Department of Orthopedic Surgery, Warren Alpert Medical School, Brown University, Providence, RI 02903, USA; 3 Department of Pathology and Laboratory Medicine, Warren Alpert Medical School, Brown University, Providence, RI 02903, USA; aamin@brownhealth.org

**Keywords:** McCune–Albright syndrome, MAS, fibrous dysplasia, FD, GNAS, skeletal structure, bone

## Abstract

McCune–Albright Syndrome (MAS) is a rare mosaic disorder caused by somatic activating mutations of the *GNAS* gene, resulting in constitutive Gsα signaling and a broad spectrum of clinical phenotypes. The syndrome typically presents with fibrous dysplasia (FD) of bone, café-au-lait macules, and endocrinopathies such as gonadotropin-independent precocious puberty, hyperthyroidism, and/or growth hormone excess. FD, which characterizes the skeletal phenotype, results in the replacement of normal bone with disorganized fibro-osseous tissue, often leading to pain, deformities, and increased risk of fractures. This review discusses the following: 1. The molecular biology of the *GNAS* locus and its relation to the pathophysiology of FD/MAS; 2. The skeletal manifestations of FD/MAS; 3. Bone biomechanics and organizational skeletal aberrations observed in FD/MAS; and 4. Current and future therapeutic strategies for patients with FD/MAS. While there is much current literature available regarding FD/MAS, this review specifically aims to outline core understandings and summarize some of the latest investigations into the genotypic and phenotypic foundations of the disorders, while shedding new light on the biomechanical aberrations observed in skeletal structure within them and comparing them to those observed in related disease processes such as osteoporosis and Paget’s disease.

## 1. Introduction

McCune–Albright syndrome (MAS; OMIM 174800) was first described in separate reports by Dr. McCune and Dr. Albright in 1937, with both reporting on a series of patients with the shared features of osteitis fibrosa, precocious puberty, endocrine dysfunction, and areas of hyperpigmentation of the skin [1,2]. In 1938, the term “polyostotic fibrous dysplasia” was coined to further describe the skeletal phenotype of fibrous accumulation filling the medullary cavities of those affected by the syndrome as a distinct clinical entity [3]. Then, over 50 years later, in 1991, Weinstein et al. made the connection between the clinical phenotype observed in MAS and activating mutations of the alpha subunit of a stimulatory G protein (Gsα), paving the way for greater understanding of the clinical manifestations, underlying pathophysiology, and potential treatment modalities for those affected by the disorder [4].

While the terms MAS and fibrous dysplasia (FD; OMIM 174800) are often used interchangeably, they are in fact distinct, yet related clinical presentations. FD describes the replacement of bone with scar-like tissue and can present as monostotic (affecting one bone) or polyostotic (affecting multiple bones). MAS refers to a subset of FD that includes associated findings such as precocious puberty, endocrine dysfunction, and areas of hyperpigmentation of the skin. According to the FD/MAS international consortium, MAS is defined as the presence of FD (monostotic or polyostotic) and one or more classic extra-skeletal manifestations, or, in the absence of FD, two or more classic extra-skeletal manifestations, such as 1. Café-au-lait skin macules with irregular borders historically referred to as resembling the “coast of Maine.” 2. Precocious puberty due to gonadotropin-independent sex steroid production; 3. Thyroid lesions; 4. Excess growth hormone production; or 5. Neonatal hypercortisolism [5].

All three of these presentations (monostotic FD, polyostotic FD, and MAS/FD) have been linked to activating mutations in the *GNAS* gene, abbreviated from its former title of the guanine nucleotide-binding protein (G protein) alpha-stimulating activity polypeptide 1 [6]. The prevalence of FD in the general population has previously been estimated to be between 1/5000 and 1/10,000 [7], though these figures have been challenged [8]. The prevalence of MAS/FD has been estimated to be between 1/100,000 and 1,000,000 [9]. Estimates in the relative prevalences of patient presentations across the FD spectrum are consistent, indicating that monostotic FD is likely the most common (representing approximately 65–75% of FD cases), followed by polyostotic FD (approximately 20–35%) and MAS (less than 5%) [10,11]. An even rarer form exists, termed Mazabraud syndrome, and is defined as the presence of FD with associated intramuscular myxomas, representing approximately 1% of those on the FD spectrum [10]. It should be noted, however, that epidemiological data on MAS/FD are an area of active investigation, and many cases of mild or asymptomatic FD may go undiagnosed or be incidentally noted on imaging studies [12,13].

In the ensuing decades since the initial connection between the clinical phenotype of MAS and its genotypic underpinnings, much has been investigated, and a rich collection of literature about the disorder and its various manifestations now exists. This report will discuss many of these findings, and in doing so, we aim to 1. Describe the structure and function of *GNAS*, the gene for which aberrations have been determined to be the cause of MAS/FD; 2. Discuss the classical skeletal phenotypes of those affected by FD/MAS; 3. Review bone biomechanics and organizational skeletal structure and explore how these are affected in FD/MAS and related disorders; and 4. Discuss current and future therapeutic tools created from the study of MAS and the *GNAS* gene.

## 2. The GNAS Gene

The human *GNAS* gene is found on the long arm of chromosome 20 in humans and encodes at least five main transcripts: the α-subunit of the heterotrimeric stimulatory G protein (Gsα), the neuroendocrine secretory protein 55 (NESP55), the extra-large variant of Gsα (XLαs), the A/B transcript, and the *GNAS* antisense transcript [14,15]. Aberrations affecting the α-subunit of the heterotrimeric stimulatory G protein (Gsα) are the causal mechanism of FD/MAS and will be the focus of this section. In 1986, it was determined that the *GNAS* locus produces at least four functional Gsα isoforms via alternative splicing [16]. Efforts investigating the roles of the proteins derived from these splice variants are ongoing. While these products have been shown to be differentially expressed in various tissues, and though at least one mutation affecting some but not all of these proteins has been linked to a form of Albright hereditary osteodystrophy and pseudohypoparathyroidism, it has been argued that there is limited functional difference between them [17,18].

The interplay between various pathologies and the genetics of *GNAS* and the Gsα subunit has proven to be a particularly rich area of investigation. Patients with maternally derived germline *GNAS* mutations have been shown to develop pseudohypoparathyroidism 1A (PHP1A) with associated obesity and hormonal resistance, while those with paternally derived germline *GNAS* mutations have been shown to develop pseudopseudohypoparathyroidism (PPHP) without associated obesity or hormonal resistance [19]. Unlike these germline-derived alterations in *GNAS* functioning, the most common mutation that leads to MAS/FD displays somatic mosaicism. Typically, in MAS, the arginine found at position 201 on the *GNAS*-derived protein is replaced by a cysteine or histidine residue early in embryogenesis, leading to impairment in its GTPase activity and subsequent inappropriate constitutive activation in the affected cells [20]. The time course of this variation is particularly important when considering the clinical implications of MAS. Because the mutation takes place during embryogenesis, rather than being passed down from the parents, the scope of bodily tissues affected can vary widely, with mutations occurring earlier in embryogenesis affecting more tissues (and thus leading to a more severe clinical phenotype) than those occurring later in embryogenesis. There is also a cell lineage-specific component to these mutations, with mutations affecting endocrine tissues leading to various endocrinopathies, those affecting skin and melanocytes leading to the distinct “coast of Maine” hyperpigmentation, and those affecting bone leading to fibrous dysplasia.

### Gsα and Stromal Stem Cell Differentiation

As we have discussed above, the *GNAS* locus encodes the stimulatory G protein alpha subunit Gsα. Increased constitutive Gsα signaling drives cell proliferation but impairs terminal differentiation of bone marrow stromal stem cells and is the underlying cause of the bone defects observed in FD and FD/MAS [21]. In a murine model in which Gsα was conditionally deleted, Wu et al. demonstrated that the lineage commitment of stromal stem cells to osteoblasts was decreased while osteogenic differentiation was accelerated, resulting in the synthesis of biomechanically incompetent matrix [22]. Examination of the tissues revealed that osteoblast progenitors were diminished and that woven, rather than lamellar trabecular, bone was produced. Several seminal conclusions can be made regarding bone matrix formation from this preclinical model of Gsα deficiency: (1) Gsα regulates bone formation by facilitating the commitment differentiation of mesenchymal progenitor cells to the osteoblast lineage and limiting the differentiation of committed osteoblasts to the production of bone with normal mechanical characteristics; (2) Deletion of Gsα in osteoprogenitors leads to decreased trabecular bone and profound osteoporosis; (3) The decreased bone mass is due to impaired bone formation rather than enhanced bone resorption; (4) The bone that is produced is largely woven bone with disorganized collagen fibrils within the matrix; and (5) Gsα deletion results in an acceleration of osteoblast differentiation into mature osteocytes while simultaneously contributing to bone pathology by reducing the number of osteoblasts contributing to the production of woven bone. Taken together, this study is part of a growing body of evidence showing Gsα plays several crucial roles in osteoblast lineage development and in normal bone development, and increased Gsα activity in fibrous dysplasia is a key abnormality resulting in abnormal bone matrix, woven bone, and skeletal pathology, including fractures.

These observations have been supported by other studies showing that while enhanced Gsα signaling accelerates the osteogenic lineage commitment of the stromal stem cells, their further differentiation into mature osteoblasts is inhibited, resulting in the formation of fibrous dysplasia bone lesions consisting of fibro-osseous matrix and woven bone [22,23]. The lesions consist of fibroblast-like cells that express early osteoblastic markers, such as alkaline phosphatase [21,24]. In Gsα deficiency, reduced Wnt signaling and the upregulation of the Wnt inhibitors sclerostin and Dickkopf-1 (DKK1) have been observed [22]. Furthermore, the presence of increased amounts of cAMP, as is seen in activating mutations of Gsα, has been shown to inhibit bone mineralization, stimulate osteoclastogenesis, induce shape derangements in osteoblasts, and dysregulate several bone matrix proteins [21]. These findings have suggested that cAMP is important in regulating osteoblastic maturation and osteoclast formation [25]. While recent literature has further described mechanisms by which stromal stem cells impact bone formation, such as through the mediation of adipocytic differentiation and influences on the local microenvironment [26], in-depth exploration of these data is beyond the scope of this review and can be found in other recently published works [27].

Taken together, the current body of literature demonstrates that Gsα signaling early in the osteoblast lineage is crucial for the formation of normal bone. In the absence of Gsα, decreased commitment of mesenchymal progenitor cells to the osteoblast lineage and pathologically accelerated osteogenic differentiation yield woven bone of substantial fragility. The clinical manifestations of these aberrations are discussed in the next section, and the proposed mechanisms by which they alter bone biomechanics and cause fractures are discussed later in this review.

## 3. Skeletal Manifestations of FD/MAS

Though FD and MAS have been researched for almost a century, the entities remain an area of active investigation, from the characterization of skeletal phenotypes to defining clinical disease spectrums and modeling pain pathways in those affected. As recently as 2025, new light has been shed on the pathophysiology of bone pain in FD [28], surgical intervention strategies [29,30], and novel therapeutic targets for FD [31,32], with the latter being discussed in greater detail at the end of this review. This section aims to explore some of the common skeletal polymorphisms associated with FD/MAS, as well as briefly review some of the latest literature investigating and further delineating patient subpopulations within these groups.

### 3.1. Appendicular Manifestations of FD/MAS

**Figure 1:**
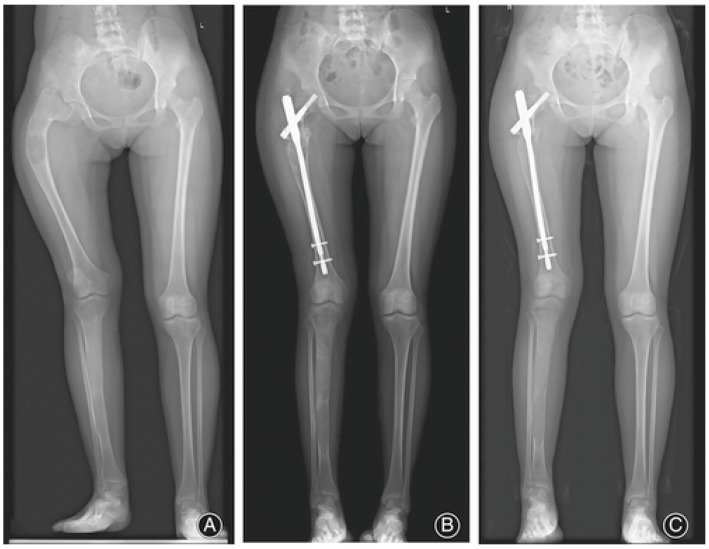
A 16-year-old patient displaying the classic coxa vara “shepherd’s crook” deformity, as well as a bone lesion in the right femur. Anterior–posterior radiographs are shown before surgical intervention (**A**), as well as 2 and 24 months postoperatively (**B**,**C**). Adapted with permission from Ref. [34]. 2022, *Orthopaedic Surgery*.

Common manifestations of FD/MAS in the appendicular skeleton include bowing and leg length discrepancies, with patients frequently presenting in childhood with gait disturbances, pain, and pathologic fractures [13]. The proximal femur is often affected, leading to the classic coxa vara presentation sometimes referred to as a “shepherd’s crook” deformity (Figure 1), though coxa valga and mixed varus/valgus deformities are also seen [13]. Patients with severe phenotypes requiring assistance with ambulation usually show signs of pathology early in life, with one longitudinal study showing that 92% of patients who eventually required ambulatory assistance showed this need by age 17, with a median age at which assistance was needed being age 7 [33]. Classification and treatment strategies of lower limb deformities in FD have been a rich area of investigation [34,35,36].

### 3.2. Axial/Craniofacial Manifestations of FD/MAS

Though some of the most classic skeletal phenotypes associated with FD/MAS are of the appendicular skeleton, those of the axial and craniofacial skeleton are well-documented as well. One study evaluating 138 patients with FD/MAS found that 61% had some degree of scoliosis, with 22% of those with scoliosis meeting criteria for a severe phenotype [37]. Regarding the pelvis and ribs, FD is the most common cause of a benign expansile rib lesion observable on radiographs, with other observable radiologic features including lytic lesions and minor calcifications [5].

**Figure 2:**
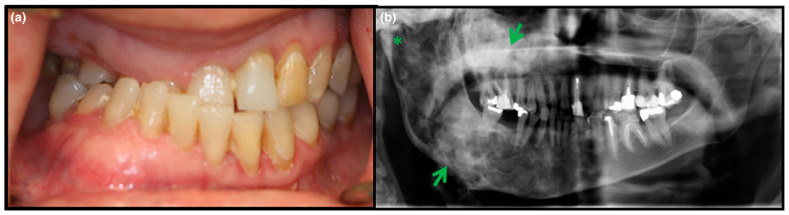
A patient with craniofacial FD. (**a**) Significant malocclusion can be observed with dental crowding and malrotation of the teeth. (**b**) Radiograph of the same patient revealing maxillary and mandibular FD (green arrows). Involvement of the mandibular condyle can also be seen (asterisk). Adapted with permission from Ref. [41]. 2017, *Oral Diseases*.

Some research indicates that the craniofacial skeleton is the most commonly affected area of the skeleton in patients with FD/MAS, with reports estimating between 10 and 25% prevalence in those with monostotic FD and between 50 and 90% prevalence in those with polyostotic FD [38,39]. One study evaluating 32 patients with FD found that 84% had involvement of the maxilla and/or mandible, with 81% having some level of dental malocclusion [40] (Figure 2). Other potential craniofacial manifestations include those of the nasal conchae, leading to nasal obstruction; those of the orbits, leading to potential visual disturbances; or those of the ossicles, which can lead to impaired hearing [41]. Many patients present with some level of bone pain, though presentations can be extremely heterogeneous. A recent study examining pain subtypes in patients with craniofacial lesions of MAS found three distinct subtypes: one characterized by severe symptomology, including frequent headaches, severe craniofacial pain, photophobia, anxiety, and depression; another characterized by milder symptoms with lower levels of pain and interference with daily activities; and a third characterized by a heterogeneous presentation with mild to severe symptoms [42].

## 4. Bone Biomechanics and Fibrous Dysplasia

**Figure 3:**
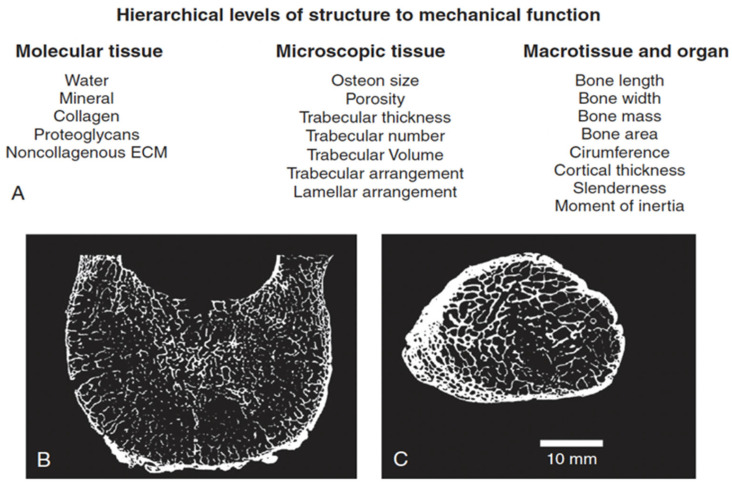
Hierarchical organization of bone. The hierarchical levels of bone composition and structure. (**A**) The primary component elements are at each of the three levels of structural hierarchy. The lower panel shows cross-sections (obtained via micro-computed tomography) of the human vertebra (**B**) and human femoral neck (**C**). Adapted with permission from Ref. [44]. 2021, *Wolters Kluwer Health, Inc*.

FD is characterized by the disorganization of bone structure on multiple hierarchical levels. As discussed earlier in this review, several reports have detailed abnormalities in bone matrix and cells as a consequence of *GNAS* mutations and resulting constitutively activated Gsα and high levels of cAMP [21,24,43]. In this section, we review the relationships of bone material and structure consequent to disorganization in FD and potentially leading to skeletal morbidity such as fractures and deformity. The structural functions of bone and its ability to meet its biomechanical demands of modulating stress depend upon its chemical and structural integrity on several multi-scale levels. FD and other disease processes affecting the skeletal system can affect some or all these levels, resulting in the inability of bone to fulfill its biomechanical functions of support. The scalar hierarchy that we consider is the biochemical matrix, the microarchitecture, and the cortex. The hierarchical organization of bone has recently been reviewed, and the relationship of structure to function and the compromise of function, namely fractures, has been detailed (Figure 3).

### 4.1. Biomechanics of Bone

**Figure 4:**
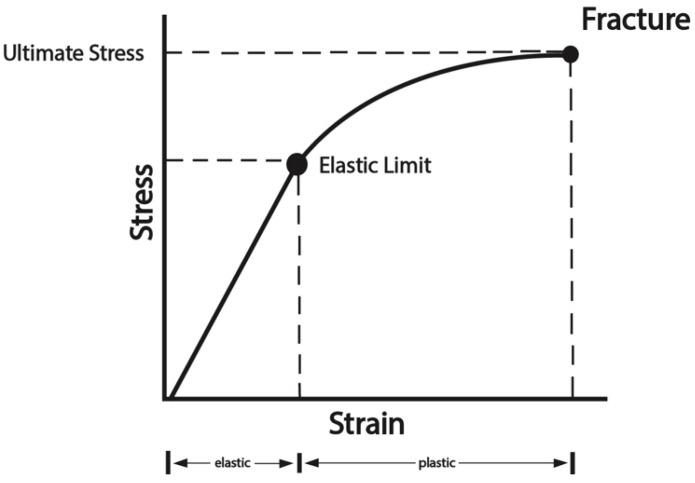
Stress–strain curve. The curve displays the strain (deformation) with applied loads (stress). The slope of the curve is termed the modulus of the bone and expresses the extent of deformation at progressive loads. The linear portion of the curve is the elastic phase, where deformation is recoverable with the release of the load. Deformation in the plastic phase is not recoverable. The elastic limit is the transition point between the two material behaviors. The ultimate stress is the point beyond which the application of further load produces a fracture. Adapted with permission from Ref. [45]. 1997, *Semin Nucl Med*.

The mechanical functions of bone can be expressed in the stress–strain curve, which relates applied forces to the deformation of bone (Figure 4). The response of bone to applied loads can be characterized as elastic or plastic [45]. In elastic deformation, bone returns to its unloaded shape after release of stress, while in plastic deformation, the deformity or strain is not recoverable, and permanent distortion can occur. The point at which plastic deformation occurs is termed the yield point. Application of load beyond the ultimate stress point can cause fracture. In gross specimens, or in in vivo situations, abnormalities in bone matrix, such as those seen in osteoporosis, Paget’s disease, and fibrous dysplasia, make the bone more sensitive to stress and failure [46]. Bone strength is determined by bone geometry, cortical thickness and porosity, trabecular bone morphology, and intrinsic material properties of bony tissue [47,48]. Fracture is largely due to decreased bone strength, but the mechanical behavior of a bone and whether it will fracture under a given loading condition is governed by the interaction between the properties of the bone material and how this material is arranged spatially [49].

#### 4.1.1. Levels of Bone Organization—Matrix-Level Organization

**Figure 5:**
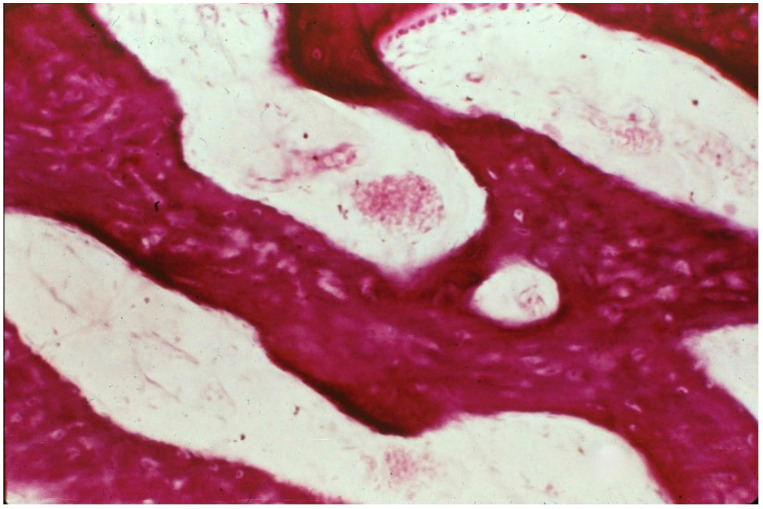
Woven bone shown with H&E stain. Trabecular bone exhibiting osteocyte lacunae but no internal organization to the osteoid matrix. Woven bone has little mechanical strength and minimal ability to carry load. Adapted with permission from Ref. [50]. 1994, *American Academy of Orthopaedic Surgeons*.

**Figure 6:**
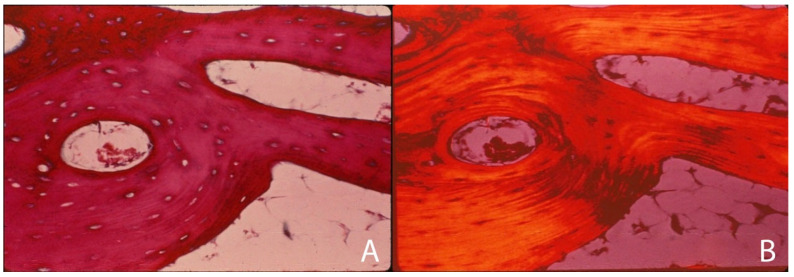
Lamellar bone shown with (**A**) H&E stain and (**B**) unstained with polarized light. (**A**) Bone matrix organized into circumferential or tubular lamellae. This organizational pattern provides the matrix with substantially greater load carriage and strength. (**B**) Polarized light image of lamellar bone, highlighting the orientation of collagen within the lamellar bone. Adapted with permission from Ref. [50]. 1994, *American Academy of Orthopaedic Surgeons*.

Bone function is an interplay of genetic and mechanical factors, each contributing substantially to the biomechanical abilities of bone. Matrix-level organization describes the way cells and the extracellular matrix are arranged within bone at the microscopic scale. The extracellular matrix of bone is composed of two components: the organic matrix, largely type 1 collagen with about 10% non-collagenous proteins, and the inorganic component of calcium hydroxyapatite. The fibers of collagen, non-collagenous proteins, and the mineral crystals precipitated within and around the fibers form a unique composite material. The organic matrix contributes to tensile strength and fracture toughness, while the mineral phase contributes to bone’s rigidity and compressive strength. Bone can be described as woven or lamellar. Woven bone is generally considered a primitive arrangement in which the matrix, including the collagen fibers, does not display organized patterns. Woven bone is seen in development and fracture repair. In non-pathologic circumstances in the adult skeleton, woven bone is a temporary or provisional tissue that is removed and replaced by more mature and organized lamellar bone. Woven bone can be seen in pathological conditions such as tumors, Paget’s disease, and fibrous dysplasia. Because of its lack of organization, woven bone is not effective in transmitting applied stresses (Figure 5). Lamellar bone, on the other hand, is highly organized into layers, or lamellae, approximately 3–5 μm in thickness, in which the collagen fibers are oriented in the same direction as the lamellae (Figure 6).

**Figure 7:**
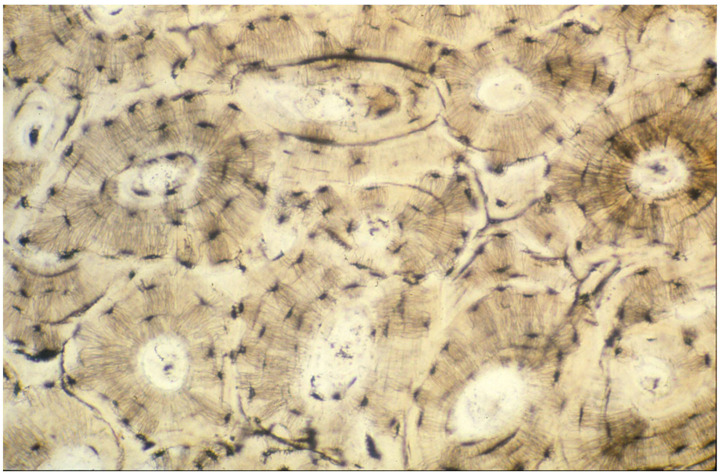
Haversian systems shown without staining. Lamellar bone organized into tubular osteons. This microarchitectural feature provides further structural strength to the matrix at the microscopic length scale. Adapted with permission from Ref. [50]. 1994, *American Academy of Orthopaedic Surgeons*.

**Figure 8:**
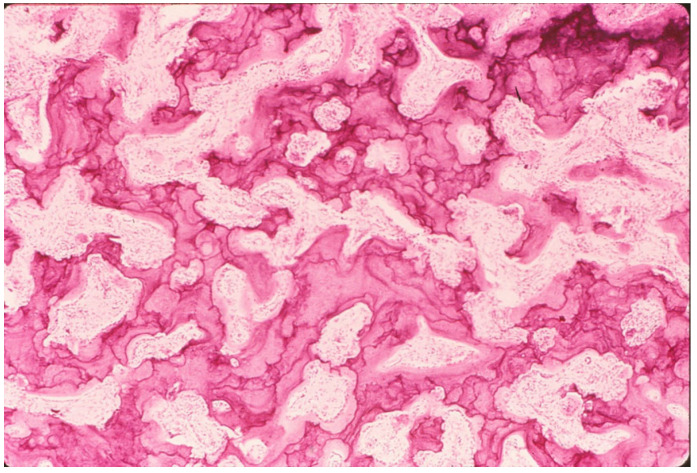
Paget’s disease shown with H&E stain. Heavy, thick trabeculae produce substantial volumetric density. However, the trabeculae have a disorganized internal structure, as shown by the chaotic arrangement of cement lines termed the “mosaic pattern.” This indicates structural weakness due to a lack of organization of the material, or matrix, of the bone. Fractures are common. Adapted with permission from Ref. [50]. 1994, *American Academy of Orthopaedic Surgeons*.

Lamellar bone is organized into Haversian systems, which undergo continual remodeling, offering another element of structural organization at the microscopic length scale (Figure 7). The importance of the organization of bone matrix is exemplified by Paget’s disease (Figure 8). Although bone thickness and volumetric density may be normal or even high, the lack of organization of the matrix reduces bone strength and leads to a propensity to fracture. Radiographically, bone may appear dense, but histologically, the organization is chaotic with cement lines in random directions, known as a mosaic pattern.

#### 4.1.2. Levels of Bone Organization—Microarchitecture

**Figure 9:**
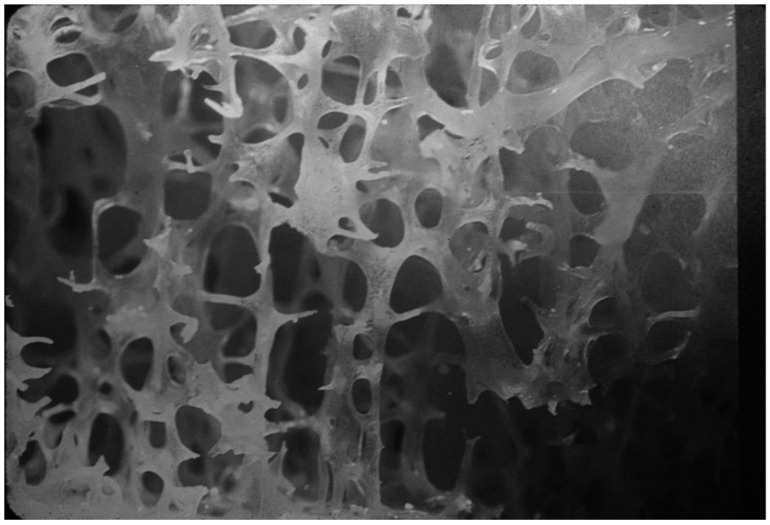
Macroscopic view of the trabecular orientation in cancellous bone. Many trabeculae are plate-like in configuration. Substantial connectivity of the trabeculae is apparent. Volumetric density is high with normal inter-trabecular spacing. Adapted with permission from Ref. [51]. 1974, *Little, Brown and Company*.

Bone microarchitecture refers to the structural organization of bone tissue, especially the arrangement and connectivity of trabeculae and the porosity of cortical bone. Bone strength is determined by numerous parameters such as overall bone geometry, cortical thickness and porosity, as well as microarchitectural features of trabecular morphology [48] (Figure 9).

**Figure 10:**
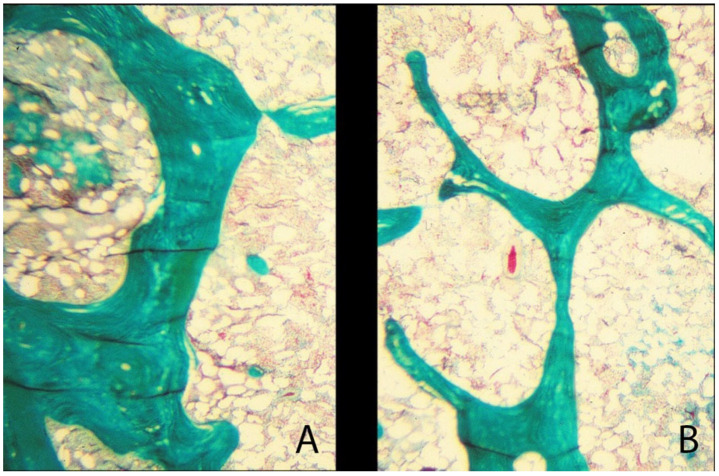
Trabecular histology shown with Goldner’s trichrome stain. (**A**) Normal trabeculae are robust in thickness with normal volumetric density, good connectivity, and the ability to transmit applied stress. (**B**) In osteoporosis, the trabeculae are thin and discontinuous with low volumetric density and wide inter-trabecular space, and therefore less ability to transmit load. Adapted with permission from Ref. [44]. 2021, *Wolters Kluwer Health, Inc*.

The mechanical properties of trabecular bone are heavily influenced by the volume fraction, or the volume of mineralized bone per unit volume of the specimen. In fact, most morphologic properties calculated from three-dimensional microcomputed tomography images, such as trabecular thickness, trabecular spacing, connectivity, and structural index, are to some extent correlated with the volume fraction [52]. Decreased bone strength is associated with decreased trabecular thickness and number, increased inter-trabecular space, modification of the trabecular shape from plates to rods, and decreased trabecular connectivity as demonstrated by histomorphometry or micro-computed tomography [53,54] (Figure 10).

**Figure 11:**
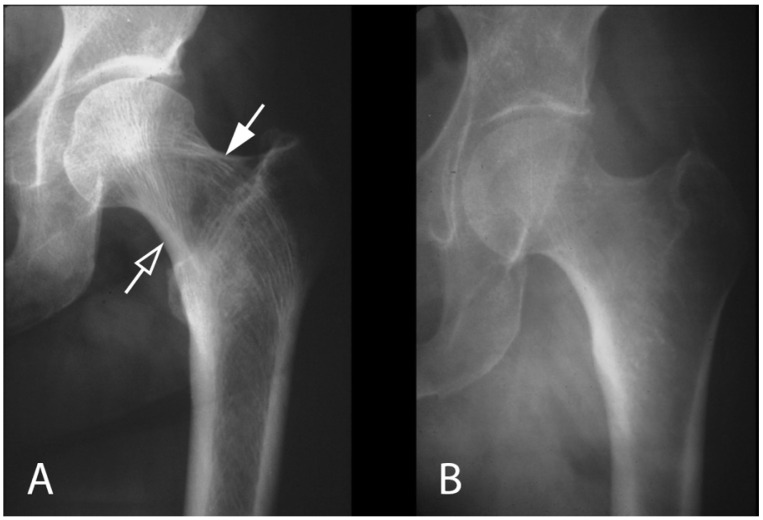
Radiographs of trabecular microarchitecture. (**A**) Normal proximal femur demonstrating compressive (open arrow) and tensile (closed arrow) trabeculae. Compressive loading is about 10° off the vertical, and the compressive trabeculae are aligned to transmit this load to the medial cortex. (**B**) In osteoporosis, the ability of osteoblasts to align trabeculae is lost, and trabeculae themselves disappear. Adapted with permission from Ref. [44]. 2021, *Wolters Kluwer Health, Inc*.

In osteoporosis, loss of trabecular volume is observed together with trabecular thinning and loss of connectivity of trabeculae, often increasing the risk of fracture. The anisotropy, or directional dependence, of trabecular bone is based upon trabecular alignment and is extremely important in providing bone with the ability to bear stress. It is another hierarchical scale in the biomechanics of bone under physiological load. Osteoblasts sense the location, orientation, and magnitude of applied load and organize bone trabeculae to best resist stress and maintain structural integrity to avoid fracture. The property of trabecular organization and stress transmission in response to applied load is expressed as Wolff’s law. Subchondral trabecular bone supports the subchondral plate against overloading from joint reactive forces and distributes that load to the cortical bone. Cortical bone resists torsional and bending loads and dissipates applied stresses. For example, trabecular bone in the proximal femur is aligned to best transmit compressive and tensile joint reactive forces from the subchondral plate to the cortices (Figure 11). Because of the offset between the center of rotation of the femoral head and the midshaft, a bending moment is experienced, and tensile trabeculae offset this stress.

#### 4.1.3. Levels of Bone Organization—Cortex

A variety of features of cortical bone contribute to its resistance to fracture. In biomechanical terms, a distinction is usually made between the mechanical behavior of bone tissue as a material and the mechanical behavior of a segment of bone as a structure. The mechanical competence of bone to resist fracture reflects intrinsic material properties (elasticity, strength, and toughness, or energy to failure) and geometry (size and shape). Structural properties of cortical bone that are commonly used as a surrogate for its mechanical strength include thickness of the cortex, cortical cross-sectional area, and area moment of inertia. Microstructural properties within the cortex, such as cortical porosity, crystallinity, and the presence of microcracks, also influence bone’s mechanical competence [55]. Structure and microarchitecture are determinant aspects of bone strength and essential elements for the assessment of bone mechanical properties. The main structural determinants of the mechanical strength of bone include width and porosity in the cortical bone, and shape, width, connectivity, and anisotropy in the trabecular bone. Microarchitecture seems to be a determinant of bone fragility independent of bone density [56].

### 4.2. Bone Pathology of Fibrous Dysplasia

As we have seen, the mechanical properties of bone are defined both by the properties of the material of which it is made, namely the bone matrix, and its structural and microarchitectural organization [57]. Diseases involving the medullary canal, such as FD, disrupt both the matrix and microstructure of bone, contributing to deformity and fracture. They are characterized by endosteal resorption resulting in medullary canal widening, often with associated periosteal bone formation, cortical thickening, and the disruption of trabecular architecture. The resulting pathomechanics involve a diminished ability to transmit applied load and a material failure (i.e., fracture) due to passing the ultimate stress point on the stress–strain curve (Figure 4).

At the matrix-level scale of FD bone tissue, the failure of completion of the lineage commitment of osteogenic progenitor cells to functional osteoblasts, associated osteoclastogenesis, and the production of a fibrous stroma all contribute to a structurally unsound fibro-osseous matrix with amorphous calcification. Rather than normal, organized lamellar bone tissue, the bone that is produced is predominantly woven bone. Bone marrow stromal cells also produce high levels of IL-6, which stimulates osteoclastogenesis. Osteoclasts increase the amount of bone resorption so that, on the scale of the bone matrix, the tissue is disorganized and chaotic, undergoing excessive resorption that is insufficient for the biomechanical function of bone as a supporting structure [13].

**Figure 12:**
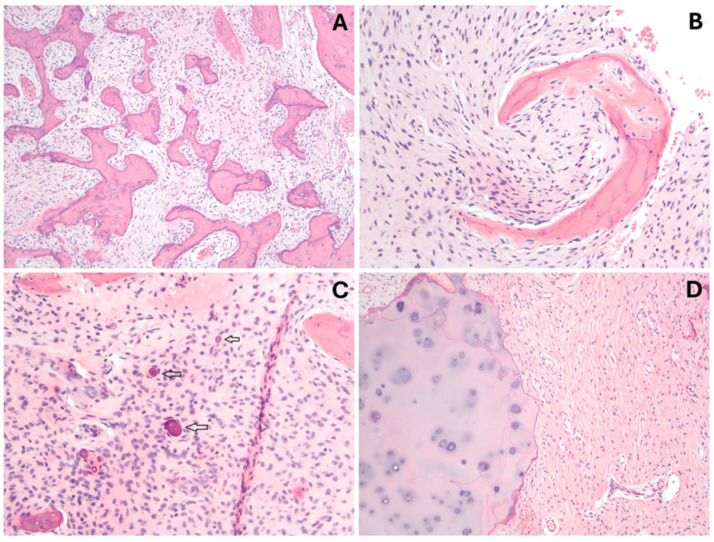
Fibrous dysplasia histology. Microscopic view of fibrous dysplasia: (**A**) Irregular curvilinear and trabecular woven bone in a bland fibroblastic background (H&E, 4×). (**B**) Curvilinear, C-shaped bone trabecula with an inconspicuous osteoblastic rim is characteristic (H&E, 10×). (**C**) Rounded, concentric (psammoma-like) calcifications are present within fibrous tissue (arrows) (H&E, 10×). (**D**) Nodules of hyaline cartilage can be seen touching fibrous tissue. These can undergo endochondral calcification (H&E, 4×).

At the microarchitectural scale, FD is characterized by inhibition of trabeculae formation and organization. The trabeculae that are formed are typically thin, discontinuous, and surrounded by abundant fibrous tissue (Figure 12). Consequently, there is a limited bone response at the microarchitectural scale to applied stress. Overall, bone and bone marrow in FD are converted into an architecturally disorganized and mechanically unsound fibro-osseous tissue [58].

**Figure 13:**
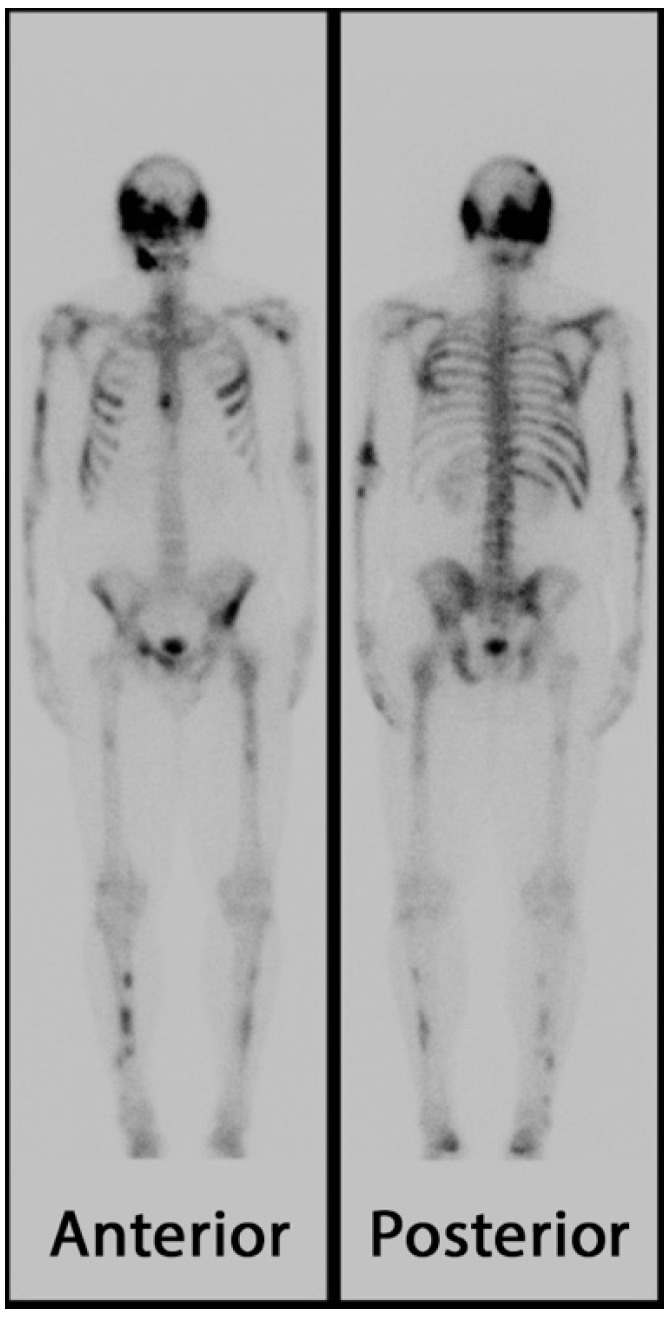
Technetium bone scan of a patient with fibrous dysplasia. Dark areas of increased uptake of the radiotracer indicate bone remodeling or fracture and are notable throughout the appendicular bones, ribs, and skull. Images displayed with patient consent.

**Figure 14:**
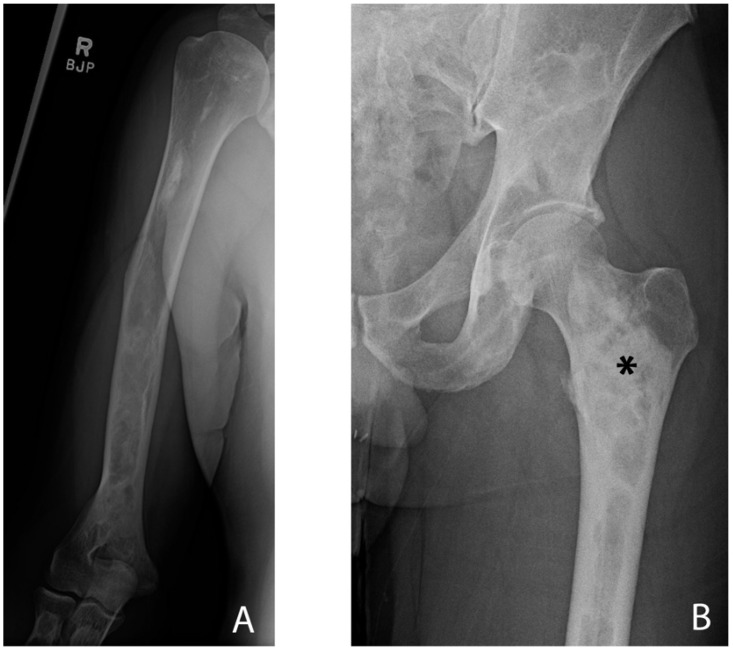
Radiographs of a patient with fibrous dysplasia. (**A**) The humerus exhibits a wide medullary canal with endosteal resorption and very thin cortices. Heterogeneous medullary patterns indicate fibro-osseous matrix with prominent lytic areas. (**B**) The proximal femur displays faint compressive trabeculae in the femoral head and neck and replacement of the trabecular bone. Replacement of the medullary canal with fibro-osseous matrix can be observed, especially in the inter- and sub-trochanteric areas (asterisk). Extensive endosteal resorption is apparent. Images displayed with patient consent.

The pathophysiology of FD contributing to structural failure of bone on multi-scale levels (matrix, cortex, trabeculae, and microarchitecture) is readily observable in radiographs, particularly in those of the appendicular skeleton. The abnormal matrix and microfractures can be seen on a technetium bone scan as enhanced uptake of the radiotracer (Figure 13). Radiographs demonstrate the abnormal matrix with areas of radiolucency and amorphous calcification, and loss of trabeculations. The matrix changes are often referred to as a “ground glass” appearance, though this is a descriptive term of the radiographs, and the matrix may exhibit regions of resorption and radiodensity representing structurally unsound fibro-osseous tissue (Figure 14). Widening of the medullary canal with thinning of the cortex and endosteal erosion are features in some bones. The overall scene, histologically and radiographically, is chaotic, with fibrous, cartilaginous, and osseous tissue in no functional order. The structural and pathomechanical result of the fibro-osseous matrix and lack of secondary scale organization is an inability to transfer load to the cortex and structural failure of bone, potentially leading to fractures and deformities.

## 5. Current and Future Therapeutics

Surgical intervention remains central for addressing structural complications of FD/MAS. Corrective osteotomies are often employed for long bone deformities, particularly in the proximal femur, where shepherd’s crook deformities are common. Internal fixation is frequently used for stabilization of fractures, and intramedullary rods are preferred over plates in weight-bearing bones to reduce the risk of hardware failure. Surgical planning is complicated by the fragile, poorly mineralized bone characteristic of FD/MAS, which increases the risk of nonunion, hardware failure, and progression of postoperative deformity. Additionally, active lesions can continue to expand even after surgical intervention, especially in the setting of uncontrolled endocrinopathies such as growth hormone excess, further emphasizing the need for individualized and cautious operative strategies. In the craniofacial skeleton, decompressive procedures may be indicated when fibrous dysplasia lesions impinge upon neural structures, such as the optic nerves or brainstem, although prophylactic surgery is generally avoided unless functional compromise is evident [5].

Pharmacological management has largely targeted bone pain and secondary complications associated with high bone turnover. Bisphosphonates, such as pamidronate and zoledronic acid, are commonly utilized and have been shown to decrease bone resorption, normalize biochemical markers of bone turnover, and provide symptomatic relief for many patients [59]. Several open-label studies and case series have demonstrated reductions in bone pain with bisphosphonate therapy, though radiologic improvements in lesion structure are less consistent. Importantly, bisphosphonates have not demonstrated a definitive ability to reverse disease progression, prevent fractures, or restore normal bone architecture. Denosumab, a monoclonal antibody targeting RANKL, has emerged as a potential second-line option for patients with refractory bone pain or those who cannot tolerate bisphosphonates [59]. While promising, denosumab is associated with significant risks, including rebound hypercalcemia and rapid lesion expansion upon discontinuation, necessitating careful patient selection and monitoring.

Management of endocrinopathies is critical to reducing skeletal and systemic morbidity in FD/MAS. In girls with gonadotropin-independent precocious puberty, aromatase inhibitors such as letrozole are often employed to suppress estrogen production and delay epiphyseal closure, helping to maximize adult height and reduce the risk of early-onset skeletal complications [60]. If aromatase inhibition is insufficient, selective estrogen receptor modulators such as tamoxifen or pure estrogen receptor antagonists such as fulvestrant may be considered to suppress estrogenic effects further. Control of other endocrinopathies, including hyperthyroidism, growth hormone excess, and phosphate wasting secondary to fibroblast growth factor 23 (FGF23) overproduction is equally critical. Hyperthyroidism and phosphate wasting can exacerbate skeletal fragility and deformity, while uncontrolled growth hormone excess can dramatically accelerate craniofacial lesion growth and increase the risk of vision and hearing loss [12].

Research efforts are increasingly focused on developing disease-modifying therapies that address the underlying molecular defects of FD/MAS. Targeting the downstream consequences of *GNAS* activation holds particular promise. Strategies under investigation include the inhibition of protein kinase A, a key mediator of cAMP signaling, and modulation of the Wnt/β-catenin pathway to promote more normal osteogenic differentiation [12]. Furthermore, therapies targeting nerve growth factor pathways are being explored as a means to control the neuropathic component of FD-associated bone pain [59]. Advances in understanding Gαs signaling and somatic mosaicism have also led to the development of improved preclinical models, facilitating the testing of novel therapeutics aimed at halting or reversing lesion progression.

Despite ongoing progress, significant challenges remain. The mosaic nature of FD/MAS results in wide variability in disease severity, complicating efforts to establish standardized treatment protocols [12]. Currently, no available therapies have been shown to halt or reverse the expansion of fibrous dysplasia lesions. Therefore, management remains primarily symptomatic and supportive. International consensus guidelines emphasize the need for early diagnosis, coordinated multidisciplinary care, and proactive management of endocrinopathies to optimize skeletal and systemic outcomes [5]. As research continues, therapies capable of modifying the natural history of FD/MAS remain a critical unmet need.

## Data Availability

No new data were created or analyzed in this study. Data sharing is not applicable to this article.

## Abbreviations

The following abbreviations are used in this manuscript:

MAS	McCune–Albright syndrome
FD	fibrous dysplasia
Gsα	alpha subunit of a stimulatory G protein
NESP55	neuroendocrine secretory protein 55
XLαs	extra-large variant of Gsα
PHP1A	pseudohypoparathyroidism 1A
PPHP	pseudopseudohypoparathyroidism
cAMP	cyclic adenosine monophosphate
FGF23	fibroblast growth factor 23
